# The impact of teaching presence on students’ online learning experience: evidence from 334 Chinese universities during the pandemic

**DOI:** 10.3389/fpsyg.2024.1291341

**Published:** 2024-06-14

**Authors:** Wen Li, Weiping Wang

**Affiliations:** ^1^School of Public Administration, Southwestern University of Finance and Economics, Chengdu, China; ^2^Institute of China Innovation and Entrepreneurship Education, Hangzhou Normal University, Hangzhou, China

**Keywords:** online learning, teaching presence, group heterogeneity, Chinese universities, online teaching

## Abstract

Teaching presence is the core role of teachers in online education and is the most promising mechanism for developing online learning communities. Drawing on the theoretical framework of teaching presence, and based on data from an online survey of university students from 334 Chinese universities, this study constructs a framework for teaching presence and compares the differences in teaching presence among different student groups, and further explores the impact of teaching presence on students’ online learning experience and its heterogeneity in terms of gender and discipline. The study finds that teaching presence includes three dimensions: instructional design and organization, facilitating interaction, and direct instruction, and there are differences among different groups. Teaching presence has a significant impact on the online learning experience, among which facilitating interaction is the most important influencing factor. Heterogeneity examination reveals that the direct instruction dimension has a greater impact on the online learning experience of female students and students in science and engineering, while the facilitating interaction dimension has a greater impact on the experience of male students and students in science and engineering. For the development of online teaching, it is necessary to help online teachers comprehensively improve teaching presence, adopt different teaching strategies and improvement measures for different student groups, and pay attention to collecting and analyzing student behavioral data for teachers to reflect on and improve teaching.

## 1 Introduction

Because of the COVID-19 pandemic, instructors had to swiftly transition to online courses. This sudden shift has prompted the rapid advancement of online teaching. However, a substantial number of online courses struggle to effectively address students’ requirements and achieve the intended course objectives (Rovai and Downey, 2010; Allen and Seaman, 2014). Scholars have undertaken extensive research to explore factors contributing to the enhancement of interaction quality within online courses. Notably, one crucial area that requires further investigation concerns students’ engagement with their online instructors, commonly referred to as teaching presence (Garrison et al., 1999; Khalid and Quick, 2016).

Teaching presence is a concept derived from the “Community of Inquiry” (COI) model, which also includes cognitive presence and social presence (Garrison et al., 1999). Three presences in COI framework are interrelated and interdependent constructs rooted in collaborative constructivist learning to guide online and blended learning (Armellini and De Stefani, 2016; Amemado and Manca, 2017). Teaching presence is the primary catalyst for formation of social presence and cognitive presence (Shea and Bidjerano, 2009) and has been interpreted as effective instructional strategies during the learning processes (Akyol et al., 2010). The work of Akyol and Garrison (2008) provides further support by highlighting the crucial role of teaching presence in facilitating student perceived learning and satisfaction. Students’ satisfaction and learning outcomes together can represent a better understanding of online learning experience. Learning outcomes measure whether students achieve competencies in their learning (Weinert, 2001). And learning satisfaction represents an attitudinal construct and measures the affective aspect (Goh et al., 2017).

China ranks first globally in terms of both the number of online Massive Open Online Courses (MOOCs) offerings and the learner population. As of November 2022, the total count of MOOCs is projected to surpass 61,900. Furthermore, there will be an estimated 402 million registered users, 979 million learners, and 352 million MOOC credits earned by current students (Ministry of Education of the People’s Republic of China, 2023). A great number of studies have revealed that teaching presence is crucial for ensuring student satisfaction in online courses. But the effects of the instructor’s teaching presence on students’ online learning experience are unclear. There is a need for continuing research studies related to specific areas, such as pedagogical strategies to promote learners’ online learning experience, the impact of learner characteristics on learner’s online learning experience (Cereijo et al., 1999; Hartley and Bendixen, 2001).

Hence, the purpose of this study is to construct a complete theoretical framework of teaching presence and explore these variables that may influence student’s satisfaction in mainland China. Concurrently, it also determines the association between teaching presence, gender, discipline and student online learning experience.

## 2 Literature review

### 2.1 Teaching presence

Teaching presence has been confirmed as the core role of online teachers and one of the most promising mechanisms for developing online learning communities. Teaching presence is the design, facilitation, and direction of cognitive and social processes for the realization of personally meaningful and educationally worthwhile learning outcomes (Anderson et al., 2001). It mainly involves three key roles: instructional design and organization, facilitating interaction, and direct instruction (Garrison and Arbaugh, 2007). Despite there are many studies on the teaching presence, its measurement framework is still to be explored. For example, Wang et al. (2012) designed teaching presence as five factors: course content organization, instructional teaching, assessment, teaching activity organization, and promoting discourse. Wang et al. (2021) investigated 408 Chinese college students and scaled teaching presence with five dimensions: design and organization, discourse facilitation, direct instruction, assessment, and technological support.

Meanwhile, existing research on the perception of teaching presence and its relationship with learners’ characteristics lacks consensus. Shea et al. (2006) found no association between learners’ characteristics (such as gender, age, employment status, distance from the campus, student registration status, reasons for taking online courses, and course duration) and their perception of teaching presence. However, others have discovered that both young (18–22 years old) and older (48–62 years old) respondents tended to equate teacher guidance and intentions with cognitive outcomes (Akyol et al., 2011). Despite the extensive research conducted on teaching presence, there is still a need for further exploration and development of its measurement framework (Rourke and Kanuka, 2009; Garrison et al., 2010).

### 2.2 The impact of teaching presence on online learning

The important impact and role of teaching presence on learners’ online learning experience have been noticed and valued by scholars. The evaluation of learning effect perception and satisfaction in the context of an online learning experience occurs at the “result” level. This level involves capturing learners’ psychological feedback and their overall assessment of the attained learning outcomes and results of the course (Liu et al., 2016). The study by Khalid and Quick (2016) found a significant positive correlation between teaching presence and learning satisfaction and can be used to predict learners’ performance in learning persistence. Szeto (2015) found through case studies that compared to social presence and cognitive presence, learners’ learning outcomes are more dependent on the performance of teaching presence. Teaching presence, as indicated by Caskurlu (2018), can influence students’ participation and engagement. But in the research conducted by Zhao and Sullivan (2017), it was observed that an increase in the level of teaching presence corresponded to a decrease in students’ participation and interaction. The effects of teaching presence on students’ online learning experience are unclear.

The validation of the influence of particular aspects of the theoretical framework regarding teaching presence on the results of online learning is yet to be confirmed. Since they only conducted surveys based on a single course and a small scale, no unified conclusions have been reached. For instance, Wang et al. (2012) found in their study on the performance of teaching presence in online courses that there is a significant correlation between the level of teaching presence and learning achievements, and learners’ learning achievements can be predicted through the performance of teaching presence. Wu et al. (2017) believe that direct teaching is the most important factor in teaching presence that affects the effectiveness of online learning, while Wang et al.’s (2012) concluded that only course content organization has a significant impact on the ability factors of learning performance. Last but not least, there is not a consensus on the effects of teaching presence on online learning experience (Wang and Liu, 2020), further research is needed in understanding and interpreting the relationship between teaching presence and student learning experience.

### 2.3 Heterogeneity in the impact of teaching presence on online learning

Previous research not only focuses on the impact of teaching presence on online learning experience but also further explores contextual factors (e.g., course length, instructor facilitation, participants’ college level) as variables that influence students’ perceived teaching presence and satisfaction. For instance, Akyol et al. (2011) found that students’ perception of teaching presence and satisfaction were higher in short-term courses than longer-term courses. In the study conducted by Epp et al. (2017), it was observed that students’ perceived learning was more pronounced in instructor-led courses and longer-duration courses. However, the effect size for facilitation was found to be even more substantial in influencing students’ perceived learning outcomes.

Past studies have also investigated that discipline and gender differences on the effects of student evaluations. In terms of learning engagement, according to the research of Lu et al. (2014), liberal arts students perceive more student-centered teaching methods and teacher-student communication than students in other disciplines. But in a recent study, Lim (2019) used sequential mixed-methods and found no significant difference in students’ teaching presence levels across soft-pure, soft-applied, hard-pure, and hard-applied disciplines. Gender also has a significant impact on indicators such as academic challenge, active cooperative learning, and richness of educational practices in learning engagement (Yang and Zhang, 2016). Considering the gender differences in learning engagement and the characteristics of disciplines, even though the vast majority of previous research has shown that both teaching presence and dimensions of teaching presence are associated with student outcomes in online learning, research on how democratic variables could impact students’ online learning experiences showed mixed results. Therefore, it is particularly important to explore the heterogeneity of the impact of teaching presence on learning experience.

In summary, the theoretical model of teaching presence is relatively mature, and small-scale surveys have been used to verify the impact of teaching presence on learning outcomes. However, since current studies have not conducted large-scale surveys, there has been no synthesis of studies that provide quantitative evidence to support the relationship between teaching presence and students’ online learning experience. Based on this, this study will be based on the theoretical model of teaching presence, using large-scale undergraduate self-assessment data from Chinese universities, to investigate the impact of teaching presence on learning experience and its heterogeneity. Specifically, three research questions addressed:

1. What are the characteristics of college students’ perception of teaching presence?
2. Does teaching presence have a significant impact on student learning experiences?
3. Are there any differences in the impact of teaching presence on online learning experience?

## 3 Data and methods

The data used in this study comes from a survey of college students’ online teaching conducted in March 2020 by the Center for Teaching and Learning Development in Xiamen University (Wang and Li, 2022; Wu and Li, 2022). The survey collected 251,929 student samples from 334 universities in Mainland China. The questionnaire covers three parts: students’ basic information, perceived teaching presence by students, online learning experience. Among them, students’ basic information includes dimensions such as gender, age, discipline, institutional level, and grade. According to the research purpose, we excluded student samples that have not participated in online learning and deleted samples with missing values in key variables (case-wise deletion method). In the end, the effective sample entering the final analysis of this study is 223,092.

Online learning experience is the dependent variable in this study. Drawing on the conceptual definitions of Liu et al. (2016), this article mainly uses two items, online learning satisfaction and online learning outcome, to depict students’ online learning experience. In the questionnaire, both are measured using a 5-point Likert scale. In the data analysis, drawing from the *refine approach* proposed by DiStefano et al. (2009), we used a regression method to create a factor scores (McNeish and Wolf, 2020), and then using the min-max normalization method, they are rescaled into 0–1 (Devlieger et al., 2016; Andersson and Yang-Wallentin, 2021), and finally they were converted into 0–100 (see Table 1).

**Table 1:** Descriptive analysis of variables.

Variable	*N*	Mean	Standard deviation	Min	Max
Online learning outcome	223,092	51.35	25.8	0	100
Online learning satisfaction	223,092	64.98	21.22	0	100
Instructional design and organization	223,092	70.63	14.27	0	100
Direct instruction	223,092	42.82	19.14	0	100
Facilitating discourse	223,092	58.78	15.5	0	100
Grade	223,092			1 = Freshman; 2 = sophomore; 3 = Junior; 4 = Senior	
Freshman	88,823	39.81			
Sophomore	70,731	31.7			
Junior	54,195	24.29			
Senior	9,343	4.19			
Institutional level	223,092	2.03		1 = research university; 2 = undergraduate colleges and universities; 3 = vocational and technical colleges	
Research Universities	4,053	1.82			
Undergraduate colleges and universities	208,975	93.67			
Vocational and technical colleges	10,064	4.51			
Discipline	223,092			1 = humanities; 2 = social science; 3 = natural science; 4 = engineering technology; 5 = medicine	
Humanities	47,018	21.08			
Social science	69,054	30.95			
Natural science	30,215	13.54			
Engineering technology	67,219	30.13			
Medicine	9,586	4.3			
Male (0 = female; 1 = male)	223,092	0.42	0.49	0	1
Public colleges and universities (0 = private; 1 = public)	223,092	0.77	0.42	0	1
Age	223,092			1 = after 80; 2 = after 90; 3 = after 00	
Post80s	608	0.27			
Post-90s	99,935	44.8			
Post-00s	122,549	54.93			
Using experience (0 = no; 1 = yes)	223,092	0.46	0.5	0	1
Training experience (0 = no; 1 = yes)	223,092	0.38	0.49	0	1

For categorical variables and 0–1 variables, the values in the third column represent percentages.

The independent variable in this study is teaching presence. Through exploratory factor analysis and confirmatory factor analysis, it is also confirmed that the teaching presence of online learning for college students in China has three dimensions, namely, instructional design and organization, direct instruction, and facilitating discourse. The measurement items and reliability and validity test results of each dimension are shown in Table 2. It is particularly worth mentioning that, because the chi-square value is very sensitive to the impact of sample size in validity testing. The chi-square test is generally only suitable for cases with a sample size of 100–300. When the sample size is large, the chi-square value often becomes large. It is easy to reject the null hypothesis at this time. Given that our sample size exceeds 220,000, it is appropriate to relax the value standards of traditional model fit indicators, such as the chi-square value and RMSEA (Wu, 2009).

**Table 2:** Reliability, validity test and measurement methods of teaching presence.

Dimension	Item	Scoring	Reliability (Cronbach’α)	Validity
Instructional design and organization	The appropriate online course content	1–5 (1 means strongly disagree, 5 means strongly agree)	0.8716	*χ*2 = 773.457;*p* = 0.046;RMSEA = 0.074; CFI = 0.988; TLI = 0.975
	Teachers’ teaching attitude			
	Teaching assistants			
	Online technical service support			
	Help students develop good online learning behavior habits			
Direct instruction	Teachers keep abreast of students’ learning status		0.9086	
	Teachers keep abreast of students’ knowledge			
	Feedback on student concerns			
	Teacher on-site guidance and supervision			
Facilitating discourse	Timeliness of teacher-student interaction		0.7792	
	Communication and collaboration among students			
	Students communicate with teachers and choose learning content according to their needs			
Overall			0.8204	

In the selection of control variables (see Table 1), this study not only includes dimensions such as gender, grade, and age that are involved in existing literature but also includes variables such as the region where the university is located, institutional level, type of university, and discipline, considering the characteristics of vertical and horizontal stratification in higher education.

## 4 Empirical results

### 4.1 Differences in teaching presence among different groups

This study measures differences in categorical variables through two methods of group comparison. The first is the T-test method used for binary variables (0–1 variables). The second is the variance test method (F-test) used for multi-classification variables. Since variance analysis cannot specifically show more detailed differences between groups, researchers further use the Bonferroni multiple testing technique for pairwise post-hoc comparisons based on variance testing. Finally, we used the effect size to measures the real strength of mean comparison by Stata command (esize, esizei).

Table 3 displays the differences in teaching presence among different student groups. Overall, the group differences in teaching presence are diverse. First, except for the facilitating discourse dimension, females perform better than males in teaching presence in the other two dimensions. In terms of training and using experience, students with online learning experience and training experience have better teaching presence. In terms of the region where the school is located, universities in the eastern region perform best in all three dimensions of teaching presence, followed by the central region, and the western region is the worst. In terms of institutional level, the higher the institutional level, the better the performance in the instructional design and organization dimension, while in the direct instruction and facilitating discourse dimensions, vocational and technical colleges perform the best, and research universities perform the worst. In terms of disciplines, in the three dimensions of instructional design and organization, direct instruction, and facilitating discourse, there is a trend that natural sciences and engineering majors perform the worst, while humanities perform the best. In terms of age, except for the facilitating discourse dimension, the other two dimensions of teaching presence show a trend: the younger the age, the better the performance. By grade, in the instructional design and organization dimension, juniors perform the best, and seniors perform the worst. In the direct instruction dimension, freshmen perform the best, and seniors perform the worst, showing a trend that the higher the grade, the worse the performance.

**Table 3:** Differences of teaching presence in different groups of students.

Dimensions (T-test)	Gender	Public universities	Using experience	Training experience	/
Instructional design and organization	2.799(*p* = 0.000; Cohen’s *d* = 0.193)	−1.498(*p* = 0.000; Cohen’s *d* = −0.109)	−1.010(*p* = 0.000; Cohen’s *d* = −0.057)	−2.376(*p* = 0.000; Cohen’s *d* = −0.150)	/
Direct instruction	2.231(*p* = 0.000; Cohen’s *d* = 0.152)	0.166(*p* = 0.086; Cohen’s *d* = −0.008)	−0.229(*p* = 0.005; Cohen’s *d* = −0.009)	−1.352(*p* = 0.000; Cohen’s *d* = −0.076)	/
Facilitating discourse	0.104 (*p* = 0.115; Cohen’s *d* = 0.015)	1.1453 (*p* = 0.000; Cohen’s *d* = 0.080)	−2.370 (*p* = 0.000; Cohen’s *d* = −0.155)	−5.557 (*p* = 0.000; Cohen’s *d* = −0.380)	/

Dimensions (One-way ANOVA test)	Region	Institutional level	Discipline	Age	Grade
Instructional design and organization	*F* = 4.20 (*p* = 0.0149; *η*^2^ = 0.0000)	*F* = 333.45; (*p* = 0.000; *η*^2^ = 0.0029)	*F* = 43.25 (*p* = 0.000; *η*^2^ = 0.0007)	*F* = 9.22; (*p* = 0.000; *η*^2^ = 0.0001)	*F* = 32.84 (*p* = 0.000; *η*^2^ = 0.0003)
Direct instruction	*F* = 35.08 (*p* = 0.000; *η*^2^ = 0.0004)	*F* = 5.17; (*p* = 0.000; *η*^2^ = 0 0.0001)	*F* = 33.87(*p* = 0.000; *η*^2^ = 0.0008)	*F* = 71.28; (*p* = 0.000; *η*^2^ = 0.0009)	*F* = 63.98 (*p* = 0.000; *η*^2^ = 0 0.0014)
Facilitating discourse	*F* = 246.87; (*p* = 0.000; *η*^2^ = 0.0020)	*F* = 47.44 (*p* = 0.000; *η*^2^ = 0 0.0003)	*F* = 16.51 (*p* = 0.000; *η*^2^ = 0.0005)	*F* = 0.40 (*p* = 0.6713; *η*^2^ = 0.0000)	*F* = 162.27(*p* = 0.000; *η*^2^ = 0.0022)

In the T test, the values represent the difference between the means of the two groups. The specific calculation method is to subtract the group with high assignment from the group with low assignment.

### 4.2 The impact of teaching presence on online learning

In this section, we will use the multiple linear regression (MLR) method to explore the impact of teaching presence on online learning experience, and then use the dominance analysis to identify the relative importance ranking of specific variables.^1^ The study found that, keeping other variables constant, for every unit increase in the dimension of instructional design and organization, learning outcome improve by 0.409 points; for every unit increase in direct instruction, learning outcome improve by 0.706 points; for every unit increase in facilitating discourse, learning outcome improve by 14.670 points (see Table 4).

**Table 4:** The influence of teaching presence on students’ online learning experience.

	Learning outcome			Learning satisfaction		
	Coeff.	Standard error	Relative importance	Coeff.	Standard error	Relative importance
Independent variable						
Instructional design and organization	0.409***	(0.049)	0.001	4.668***	(0.033)	0.0546
Direct instruction	0.763***	(0.048)	0.0009	0.902***	(0.033)	0.0019
Facilitating discourse	14.670***	(0.052)	0.2747	15.454***	(0.035)	0.452
Control variable						
Male	2.622***	(0.101)		0.130*	(0.069)	
Grade (reference group: freshman)						
Sophomore	1.098***	(0.119)		0.018	(0.081)	
Junior	1.164***	(0.162)		1.066***	(0.110)	
Senior	3.452***	(0.266)		2.332***	(0.180)	
Institutional level (reference group: research universities)						
Undergraduate colleges and universities	0.141	(0.348)		−3.382***	(0.236)	
Vocational and technical college	0.893**	(0.409)		−4.625***	(0.277)	
Disciplines (reference group: humanities)						
Social science	−0.791***	(0.131)		−0.427***	(0.089)	
Natural science	−0.729***	(0.165)		−0.338***	(0.112)	
Engineering technology	−1.300***	(0.140)		−0.268***	(0.095)	
Medicine	−4.041***	(0.250)		−0.930***	(0.170)	
Public universities	−1.264***	(0.114)		−0.152**	(0.077)	
Age (reference group: post-80s)						
Post-90s	−3.586***	(0.887)		−0.474	(0.602)	
Post-00s	−3.431***	(0.891)		0.537	(0.604)	
Using experience	2.042***	(0.096)		0.084	(0.065)	
Training experience	1.860***	(0.099)		2.245***	(0.067)	
Constant	52.974***	(0.967)		67.333***	(0.656)	
Number	223,092			223,092		
*R* square	0.286			0.514		

***p < 0.01, **p < 0.05, *p < 0.1.

Continuing to examine from the perspective of learning satisfaction (Table 4), this study also confirms that teaching presence can significantly affect students’ experiences of online learning. In the satisfaction model, keeping other variables constant, for every unit increase in the dimension of instructional design and organization, learning outcome improve by 4.668 points; for every unit increase in the direct instruction dimension, learning satisfaction improves by 0.902 points; for every unit increase in the facilitating discourse dimension, learning satisfaction improves by 15.454 points.

In the control variable section, regardless of whether it is the learning outcome or satisfaction model, males have higher evaluations than females. The higher the grade, the higher the student’s evaluation. Public universities have lower evaluations than private colleges. Students with online learning experience have better effects and satisfaction with online learning. Humanities and social sciences students have better online learning outcome and satisfaction than science, engineering, and medical majors. In addition, students’ online learning experiences also show differences in other dimensions. For example, in terms of institutional level, in the learning outcome model, the higher the institutional level, the better the learning outcome. However, in the satisfaction model, the higher the institutional level, the lower the student’s satisfaction. In terms of grade, in the learning outcome model, the lower the grade, the worse the effect; while in the satisfaction model, there is no difference between students of different ages. In terms of online learning using experience, in the learning outcome model, students with using experience have higher evaluations; but in the satisfaction model, there is no significant relationship between using experience and satisfaction.

Although the above has confirmed that teaching presence will significantly improve the outcome and satisfaction of online learning, further analysis is needed on the impact of specific dimensions on effects and satisfaction, as well as the relative importance ranking in learning outcome and satisfaction. This is because the size of each coefficient cannot be used for direct comparison. Traditional practices are stepwise regression, significance testing, and coefficient standardization techniques. However, the order of introducing explanatory variables in stepwise regression is very subjective, and coefficient standardization does not know the relative importance of each dimension. Therefore, this study will introduce the Dominance Analysis proposed by Israeli (2007). This method aims to compare pairs of predictors across all subsets of the predictors in a model to determine the additional contribution that each predictor makes to the prediction model. In fact, the contribution to the coefficient of determination also reflects the contribution of different explanatory variables to the variance of the dependent variable.

In columns 3 and 6 in Table 4, the study also found that whether in the learning outcome or satisfaction model, facilitating discourse is the most important influencing factor, followed by instructional design and organization, and direct instruction dimensions. Moreover, relatively speaking, the importance of facilitating discourse is greater in the satisfaction model. Specifically, in the learning outcome model, the contribution of the facilitating discourse dimension is 0.274, which can be interpreted as the marginal contribution of this variable to the goodness of fit is 0.274. That is to say, in this linear regression, relative to the dimensions of instructional design and organization and direct instruction, the facilitating discourse dimension has a stronger explanatory power for the variance change of the dependent variable online learning outcome. In the learning satisfaction model, the contribution of the facilitating discourse dimension is 0.452. Therefore, in this linear regression, relative to the dimensions of instructional design and organization and direct instruction, the facilitating discourse dimension still has a stronger explanatory power for the variance change of the dependent variable online learning outcome.

In summary, the empirical results indicate that teaching presence has a significant impact on the online learning experience. Among the dimensions of teaching presence, facilitating discourse is the most critical factor affecting both learning outcomes and satisfaction. The study also highlights the importance of instructional design and organization as well as direct instruction. The results can be used to guide the development of online education, especially in understanding the different dimensions of teaching presence and their relative importance in enhancing the online learning experience.

### 4.3 Heterogeneity of the impact of teaching presence on online learning: gender and discipline

In this part, we will use the multiple linear regression and interactions in regression to the examine whether the relationship between the teaching presence and the online learning experience changes depending on the value of another independent variable (e.g., gender and discipline). Among the initial characteristics of learners such as student gender, grade, age, discipline, region, and institutional level, only gender and discipline show significance in the impact of teaching presence on online learning experience (see Table 3). Combining the characteristics of female learning investment and discipline mentioned in the literature review section, the researchers will explore the group heterogeneity of the impact of teaching presence on online learning experience from the perspectives of gender and discipline.

Gender heterogeneity of the impact of teaching presence on online learning is shown in Table 5. The examination of heterogeneity mainly focuses on the value and direction of the main effects and interaction terms. In the learning outcomes model, the study found that, on the one hand, the direct instruction dimension has opposite effects on females and males. Specifically, for females, for every unit increase in direct instruction, learning outcomes improve by 1.717 points. For males, for every unit increase in direct instruction, learning outcomes decrease by 0.27 points (1.717–1.987). On the other hand, the facilitating discourse dimension has a greater impact on males. Specifically, for females, for every unit increase in facilitating discourse, learning outcomes improve by 13.737 points. For males, for every unit increase in the facilitating discourse dimension, learning outcomes improve by 15.540 points (13.737 + 1.803).

**Table 5:** Group heterogeneity of the effect of teaching presence on students’ online learning.

	Gender			Discipline	
	Learning outcome	Learning satisfaction		Learning outcome	Learning satisfaction
Instructional design and organization	0.362***	4.530***	Instructional design and organization	0.031***	0.310***
	(0.068)	(0.046)		(0.005)	(0.003)
Male	2.566***	0.117*	Humanities and social sciences	1.649***	0.726*
	(0.101)	(0.069)		(0.609)	(0.414)
Instructional design and organization*male	−0.056	0.226***	Instructional design and organization*humanities and social sciences	−0.001	0.007
	(0.098)	(0.067)		(0.006)	(0.004)
Direct instruction	1.717***	1.239***	Direct instruction	0.040***	0.048***
	(0.065)	(0.044)		(0.003)	(0.002)
Direct instruction*male	−1.987***	−0.630***	Direct instruction*humanities and social sciences	−0.006	−0.009***
	(0.098)	(0.066)		(0.005)	(0.003)
Facilitating discourse	13.737***	14.844***	Facilitating Discourse	0.858***	0.907***
	(0.071)	(0.048)		(0.004)	(0.003)
Facilitating discourse*male	1.803***	1.233***	Facilitating discourse*humanities and social sciences	−0.007	−0.012***
	(0.103)	(0.070)		(0.006)	(0.004)
Control variable	Yes	Yes	Control variable	Yes	Yes
Constant	52.999***	67.371***	Constant	−2.768***	−10.208***
	(0.965)	(0.655)		(1.060)	(0.719)
Number	223,092	223,092	Number	223,092	223,092
*R* square	0.288	0.515	*R* square	0.284	0.513

***p < 0.01, **p < 0.05, *p < 0.1; Control variables: (1) For gender heterogeneity, the control variables are: age, discipline, institutional level, grade, age, university location, type of university, institutional level. (2) For discipline heterogeneity, the control variables are: age, gender, school level, grade, age, institutional location, type of university, institutional level.

In the learning satisfaction model, the study also found that the impact of teaching presence on online learning varies by gender. First, instructional design and organization have a greater impact on males. Specifically, for females, for every unit increase in instructional design and organization, learning satisfaction improves by 4.530 points. For males, for every unit increase in the instructional design and organization dimension, learning satisfaction improves by 4.756 points (4.530 + 0.226). Secondly, direct instruction has a greater impact on females. Specifically, for females, for every unit increase in instructional design and organization, learning satisfaction improves by 1.239 points. For males, for every unit increase in direct instruction, learning satisfaction improves by 0.609 points (1.239–0.630). Finally, facilitating discourse has a greater effect on males. Specifically, for females, for every unit increase in the facilitating discourse dimension, learning satisfaction improves by 14.844 points. For males, for every unit increase in direct instruction, learning satisfaction improves by 16.077 points (14.844 + 1.233).

Regarding the discipline heterogeneity of the impact of teaching presence on online learning, for ease of interpretation of the results, the five-category discipline is converted into two categories: humanities and social sciences and science and engineering. Table 5 reports the results of the discipline heterogeneity of the impact of teaching presence on online learning. In the learning outcomes model, there is no significant difference between arts and science and engineering in the impact of teaching presence. In the satisfaction model, there is a significant difference between arts and science and engineering in the impact of teaching presence. On the one hand, direct instruction has a greater impact on science and engineering. Specifically, for science and engineering students, for every unit increase in direct instruction, learning satisfaction improves by 0.048 points. For humanities and social sciences majors, for every unit increase in direct instruction, learning satisfaction improves by 0.039 points (0.048–0.009). On the other hand, the facilitating discourse dimension also has a greater effect on science and engineering students. Specifically, for science and engineering students, for every unit increase in the facilitating discourse dimension, learning satisfaction improves by 0.907 points. For humanities and social sciences majors, for every unit increase in direct instruction, learning satisfaction improves by 0.895 points (0.907–0.012).

In conclusion, this section of the study reveals that the impact of teaching presence on online learning experience varies among different groups, particularly in terms of gender and discipline. For gender, the study found that the dimensions of teaching presence have different impacts on males and females. Specifically, direct instruction is more beneficial for females, while facilitating discourse is more beneficial for males in terms of learning outcomes. In terms of learning satisfaction, instructional design and organization have a greater impact on males, while direct instruction has a greater impact on females, and facilitating discourse has a greater effect on males.

For discipline, the study did not find significant differences in the impact of teaching presence on learning outcomes between humanities and social sciences and science and engineering. However, in terms of learning satisfaction, direct instruction and facilitating discourse have a greater impact on science and engineering students compared to humanities and social sciences students.

## 5 Discussion and implications

### 5.1 Discussion

This study found that the teaching presence model is applicable to online teaching and learning in Chinese universities, consistent with previous research findings (Garrison and Arbaugh, 2007). For instance, our data analysis revealed that teaching presence positively influences student satisfaction and learning outcomes, corroborating established literature on the subject. Additionally, this study verified that teaching presence comprises three dimensions, a finding that has been subject to debate in past literature (Hoskins and Van Hooff, 2005; Shea et al., 2006). By conducting rigorous statistical analyses and quantitative assessments, we were able to confirm the distinct roles of design, facilitation, and direct instruction in shaping teaching presence, thereby providing empirical support for this conceptual framework. Moreover, our findings further revealed significant differences in online teaching activities among various student groups, challenging existing research conclusions (Piccoli et al., 2001; Arbaugh and Hwang, 2006). For example, while previous studies may have suggested uniformity in online teaching effectiveness across demographic groups (Zhao and Sullivan, 2017; Caskurlu, 2018), our data indicate that females generally outperform males, and students with prior usage experience and training exhibit stronger teaching presence. Additionally, our study uncovered regional and institutional disparities in teaching presence performance. Contrary to prevailing assumptions, universities in the eastern region demonstrated superior performance in online teaching activities. Furthermore, our analysis revealed variations in instructional design effectiveness across institutional types, with higher-level institutions excelling in this aspect, while vocational and technical colleges demonstrated strengths in direct instruction and facilitating discourse. Lastly, our findings underscored the significant impact of student discipline, age, and grade on teaching presence. Discipline-specific differences were observed, highlighting the importance of developing tailored approaches to adapt teaching presence strategies to accommodate diverse learner characteristics. By incorporating these detailed data examples and comparisons with existing research, this paper strengthens its arguments and provides a more comprehensive understanding of the implications of the findings for educational practice and future research.

Furthermore, the study results revealed that the facilitating discourse dimension is the most important influencing factor, impacting teaching presence on the online learning experience. This outcome contrasts with the findings of other previously published investigations (Qiao et al., 2021) in China based on small-scale investigations (Wang et al., 2012). In the satisfaction model, the facilitating discourse dimension holds greater importance. Although Chen et al. (2020) examined learning outcomes, their conclusions align with this article, emphasizing that social interaction is the most significant factor in online learning. However, it should be noted that this conclusion conflicts with research also based on surveys of Chinese university students (Li and Jiang, 2009). In their study, the direct instruction dimension emerges as the most important influencing factor. Our findings present a different portrait of Chinese students, often characterized as passive, reproductive, and surface learners in the literature (Jones, 1999).

This study showed that teaching presence has a significant heterogeneous impact on college students’ online learning outcome and satisfaction. Among students from different genders and disciplines, different dimensions of teaching presence have different impacts on students’ learning experiences. The direct instruction dimension has a greater impact on the online learning outcome and satisfaction of females, while the facilitating discourse dimension has a greater impact on the online learning outcome and satisfaction of males. As Qin et al. (2022) found in online learning, males pay more attention to the external environment, while females pay more attention to the effectiveness and quality of learning. In the learning outcomes model, there is no disciplinary heterogeneity in the impact of teaching presence on online learning. However, in the learning satisfaction model, there is heterogeneity, and both direct instruction and facilitating discourse dimensions have a greater impact on the learning satisfaction of science and engineering students. The findings of this study were supported by a previous study in China (Lu et al., 2014). In summary, the study concludes that teaching presence, especially the facilitating discourse dimension, is crucial in online learning experiences. There are also significant differences in how teaching presence affects different groups, especially in terms of gender and discipline.

The findings of this study contribute significantly to the understanding of online teaching and learning dynamics, particularly within the context of Chinese universities. The dominance of the facilitating discourse dimension as the most influential factor impacting online learning experiences underscores the critical role of interaction and collaboration in virtual learning environments, aligning with contemporary pedagogical theories emphasizing social constructivism and collaborative learning in online education. However, the divergence from previous research conducted in China suggests the need for contextualized investigations considering cultural factors and institutional contexts, which may shape students’ preferences and experiences regarding online interaction (Li and Jiang, 2009). Moreover, the gender and disciplinary heterogeneity in the impact of teaching presence on online learning outcomes and satisfaction reveal complex dynamics warranting further exploration. The observed differential effects among male and female students suggest underlying cognitive and socio-cultural factors influencing their engagement with online learning environments (Chen et al., 2020). Similarly, disciplinary variations (e.g., hard-applied disciplines vs. soft disciplines) underscore the importance of tailoring instructional approaches to specific requirements and conventions of different academic disciplines, highlighting the significance of discipline-specific pedagogical strategies in online education (Lim and Richardson, 2022).

### 5.2 Implications

Digital transformation is a slow process in education, which became an urgent topic in the spring of 2020 due to COVID-19 (Bogdandy et al., 2020). Our findings contribute to the emerging literature on the teacher presence framework and provide insight into online education satisfaction, potentially setting further directions for research and practice. This study has constructed a localized teaching presence guiding framework as a breakthrough point to comprehensively assist teachers conducting online teaching in improving teaching quality and satisfaction.

For educators, the model of teaching presence offers a theoretical basis for strategic choices when transitioning to online teaching roles, validating these strategies through large-scale empirical surveys. This validation is crucial for educators’ readiness for the digital transformation of education (Tóth et al., 2022). The teaching presence framework serves as a means to comprehend online learning and teaching methods, with its three dimensions offering both theoretical and practical guidance for strengthening teaching strategies in online environments. Our findings also underscore the importance for teachers to consider disciplinary and gender characteristics, advocating for the adoption of differentiated teaching strategies and improvement measures tailored to diverse student groups.

Institutions would benefit greatly from exploring ways to evaluate online learning. Our research results demonstrate that teaching presence has varying impacts on learners’ outcomes and satisfaction across different genders and disciplines. This underscores the necessity for educational institutions and educators to acknowledge these differences and adapt their strategies accordingly to enhance the online learning experience for all students. Moreover, the data sources utilized in this study are essential for future AI-powered automation (Zarifis and Efthymiou, 2022), providing valuable references for teachers on improving student learning outcomes and supporting universities undergoing digital transformation.

Governments must recognize that in online teaching at central and western universities, teachers may tend to replicate traditional face-to-face teaching practices without fully transitioning from knowledge disseminators to designers and facilitators. Addressing the regional disparities in teaching presence is crucial, with governments needing to prioritize and support the perception of teaching presence in online teaching, particularly for students from central and western universities.

### 5.3 Limitations of the study

Certainly, this study still has third areas of deficiency awaiting further advancements in subsequent research. Firstly, dominance analysis, while useful for determining predictor importance in regression models, has limitations including assumptions of linearity and challenges in interpretation. To supplement it, machine learning techniques like feature importance from random forests (Mizumoto, 2023), permutation importance, SHAP values, and LIME can offer more flexibility and robustness, especially in handling non-linear relationships and high-dimensional data. Secondly, regarding the significant gender and subject heterogeneity in the impact of teaching presence on the online learning experience, this study aims to provide a theoretical foundation by reviewing the literature on gender learning and subject characteristics. This explanation partially accounts for the important moderating role of gender and discipline. However, it is acknowledged that the current data structure limits the depth of quantitative research to uncover detailed internal mechanisms, concrete examples or case stud, and microscopic effects. While qualitative research could serve as a complementary approach, its expanded discussion is beyond the scope of this article, considering practical constraints related to article length and focus. Finally, future research should consider conducting comparisons between different countries or cultures to examine the heterogeneity of the impact of teaching presence on students’ online learning. Especially noteworthy are the differences between Confucian-heritage culture and the Western university classroom teaching model (Biggs, 1998; Karjanto and Simon, 2019). These comparisons are crucial for understanding the integration of global online learning.

## Data availability statement

The original contributions presented in the study are included in the article/supplementary material, further inquiries can be directed to the corresponding author.

## Ethics statement

Ethical approval was not required for the study on human participants in accordance with the local legislation and institutional requirements. The studies were conducted in accordance with the local legislation and institutional requirements. Written informed consent for participation was not required from the participants or the participants’ legal guardians/next of kin in accordance with the national legislation and institutional requirements.

## Author contributions

WL: Writing – review & editing, Writing – original draft, Funding acquisition, Data curation, Conceptualization. WW: Writing – original draft, Software, Methodology, Formal analysis.

## Footnotes

^1^To fully check the five main assumptions underlying multiple regression models (Osborne and Waters, 2019), we used the Stata command (e.g., estat imtest, white; estat vif) to check whether the assumptions of MLR is satisfied. This method is also used in section 4.3. We found that the assumptions of statistical test are basically met.

## References

[ref1] Akyol Z. Garrison D. R. (2008). The development of a community of inquiry over time in an online course: understanding the progression and integration of social, cognitive and teaching presence. Online Learn. 12, 3–22. doi: 10.24059/olj.v12i3.72

[ref2] Akyol Z. Ice P. Garrison R. Mitchell R. (2010). The relationship between course socio-epistemological orientations and student perceptions of community of inquiry. Internet High. Educ. 13, 66–68. doi: 10.1016/j.iheduc.2009.12.002

[ref3] Akyol Z. Vaughan N. Garrison D. R. (2011). The impact of course duration on the development of a community of inquiry. Interact. Learn. Environ. 19, 231–246. doi: 10.1080/10494820902809147, PMID: 38770804

[ref4] Allen I. E. Seaman J. (2014). Grade change: Tracking online education in the United States. Available at:http://www.onlinelearningsurvey.com/reports/gradechange.pdf

[ref5] Amemado D. Manca S. (2017). Learning from decades of online distance education: MOOCs and the community of inquiry framework. J. E-Learn. Know. Soc. 13, 21–32. doi: 10.20368/1971-8829/1339

[ref6] Anderson T. Liam R. Garrison D. R. Archer W. (2001). Assessing teaching presence in a Computer Conferencing Context. Online Learn. 5. doi: 10.24059/olj.v5i2.1875

[ref7] Andersson G. Yang-Wallentin F. (2021). Generalized linear factor score regression: a comparison of four methods. Educ. Psychol. Meas. 81, 617–643. doi: 10.1177/0013164420975149, PMID: 34262222 PMC8243200

[ref8] Arbaugh J. B. Hwang A. (2006). Does “teaching presence” exist in online MBA courses? Internet High. Educ. 9, 9–21. doi: 10.1016/j.iheduc.2005.12.001

[ref9] Armellini A. De Stefani M. (2016). Social presence in the 21st century: an adjustment to the community of inquiry framework. Br. J. Educ. Technol. 47, 1202–1216. doi: 10.1111/bjet.12302

[ref10] Biggs J. (1998). Learning from the Confucian heritage: so size doesn’t matter? Int. J. Educ. Res. 29, 723–738. doi: 10.1016/S0883-0355(98)00060-3

[ref11] Bogdandy B. Tamas J. Toth Z. (2020). Digital transformation in education during COVID-19: a case study. In 2020 11th IEEE international conference on cognitive infocommunications (CoginfoCom), 000173–000178.

[ref12] Caskurlu S. (2018). Confirming the subdimensions of teaching, social, and cognitive presences: a construct validity study. Internet High. Educ. 39, 1–12. doi: 10.1016/j.iheduc.2018.05.002

[ref13] Cereijo M. V. P. Young J. Wilhelm R. W. (1999). Factors facilitating learner participation in asynchronous web-based courses. J. Comput. Teach. Educ. 18, 32–39.

[ref14] Chen T. Gong Y. X. Pu Y. (2020). Exploring socialized meaning: interaction in online teaching and its impact on learning outcomes: based on a survey of online teaching in 334 universities. High. Edu. Res. 41, 72–81.

[ref15] Devlieger I. Mayer A. Rosseel Y. (2016). Hypothesis testing using factor score regression: a comparison of four methods. Educ. Psychol. Meas. 76, 741–770. doi: 10.1177/0013164415607618, PMID: 29795886 PMC5965529

[ref9001] DiStefano C. Zhu M. Mîndrilă D. (2009). Understanding and using factor scores: considerations for the applied researcher. Pract. Assess. Res. Eval. 14.

[ref16] Epp C. D. Phirangee K. Hewitt J. (2017). Student actions and community in online courses: the roles played by course length and facilitation method. Online Learn. J. 21, 53–77. doi: 10.24059/olj.v21i4.1269

[ref17] Garrison D. R. Anderson T. Archer W. (1999). Critical inquiry in a text-based environment: computer conferencing in higher education. Internet High. Educ. 2, 87–105. doi: 10.1016/S1096-7516(00)00016-6

[ref18] Garrison D. R. Arbaugh J. B. (2007). Researching the community of inquiry framework: review, issues, and future directions. Internet High. Educ. 10, 157–172. doi: 10.1016/j.iheduc.2007.04.001, PMID: 38606363

[ref19] Garrison D. R. Cleveland-Innes M. Fung T. S. (2010). Exploring causal relationships among teaching, cognitive and social presence: student perceptions of the community of inquiry framework. Internet High. Educ. 13, 31–36. doi: 10.1016/j.iheduc.2009.10.002

[ref20] Goh C. F. Leong C. M. Kasmin K. Hii P. K. Tan K. (2017). Students’ experiences, learning outcomes and satisfaction in e-learning. J. E-Learn. Know. Soc. 13, 117–128. doi: 10.20368/1971-8829/144

[ref21] Hartley K. Bendixen L. D. (2001). Educational research in the internet age: examining the role of individual characteristics. Educ. Res. 30, 22–26. doi: 10.3102/0013189X030009022, PMID: 38706570

[ref9002] Hoskins S. L. Van Hooff J. C. (2005). Motivation and ability: which students use online learning and what influence does it have on their achievement? Br. J. Educ. Technol. 36, 177–192.

[ref22] Israeli O. (2007). A Shapley-based decomposition of the R-square of a linear regression. J. Econ. Inequal. 5, 199–212. doi: 10.1007/s10888-006-9036-6

[ref23] Jones A. (1999). The Asian learner: An overview of approaches to learning. Melbourne: The University of Melbourne.

[ref24] Karjanto N. Simon L. (2019). English-medium instruction Calculus in Confucian-heritage culture: flipping the class or overriding the culture? Stud. Educ. Eval. 63, 122–135. doi: 10.1016/j.stueduc.2019.07.002, PMID: 38771727

[ref25] Khalid M. N. M. Quick D. (2016). Teaching presence influencing online Students' course satisfaction at an institution of higher education. Int. Educ. Stud. 9:62. doi: 10.5539/ies.v9n3p62

[ref26] Li H. Z. Jiang G. Z. (2009). Evaluation of teaching presence in online teaching: a case study of the online course 'Research methods in distance education. China Dis. Edu. 12, 44–47.

[ref27] Lim J. (2019). Disciplinary differences in a community of inquiry. West Lafayette: Purdue University.

[ref28] Lim J. Richardson J. C. (2022). Considering how disciplinary differences matter for successful online learning through the Community of Inquiry lens. Comp. Educ. 187:104551. doi: 10.1016/j.compedu.2022.104551

[ref29] Liu B. Zhang W. L. Jiang Y. J. (2016). Online course learning experience: connotation, development, and influencing factors. China Educ. Technol. 10, 90–96.

[ref30] Lu G. S. Peng Z. X. Hu W. (2014). Analysis of differences in learning experience of college students in different subjects. J. Soochow Univ. 2, 64–73.

[ref31] McNeish D. Wolf M. G. (2020). Thinking twice about sum scores. Behav. Res. Methods 52, 2287–2305. doi: 10.3758/s13428-020-01398-0, PMID: 32323277

[ref32] Ministry of Education of the People’s Republic of China, Technology empowers education and shares university resources. (2023) Available at:http://www.moe.gov.cn/jyb_xwfb/xw_zt/moe_357/2023/2023_zt01/mtbd/202302/t20230212_1043924.html

[ref33] Mizumoto A. (2023). Calculating the relative importance of multiple regression predictor variables using dominance analysis and random forests. Lang. Learn. 73, 161–196. doi: 10.1111/lang.12518

[ref34] Osborne J. W. Waters E. (2019). Four assumptions of multiple regression that researchers should always test. Pract. Assess. Res. Eval. 8:2. doi: 10.7275/r222-hv23

[ref35] Piccoli G. Ahmad R. Ives B. (2001). Web-based virtual learning environments: a research framework and a preliminary assessment of effectiveness in basic IT skills training. MIS Q. 25, 401–426. doi: 10.2307/3250989

[ref36] Qiao W. F. Liu W. T. Li M. L. (2021). Online teaching in the eyes of students: behavior, effects, and challenges - based on a survey of Tsinghua University Students' online learning behavior during the COVID-19 pandemic. Tsinghua Univ. Educ. Res. 42, 57–66.

[ref37] Qin H. X. Fang F. Zhou J. H. (2022). Gender differences in university Students' satisfaction and continuous use intention in online teaching. Univ. Educ. Sci. 1, 44–53.

[ref38] Rourke L. Kanuka H. (2009). Learning in communities of inquiry: a review of the literature. J. Dist. Educ. 23, 19–48.

[ref39] Rovai A. P. Downey J. R. (2010). Why some distance education programs fail while others succeed in a global environment. Internet High. Educ. 13, 141–147. doi: 10.1016/j.iheduc.2009.07.001

[ref40] Shea P. Bidjerano T. (2009). Community of inquiry as a theoretical framework to foster “epistemic engagement” and “cognitive presence” in online education. Comp. Educ. 52, 543–553. doi: 10.1016/j.compedu.2008.10.007

[ref41] Shea P. Li C. S. Pickett A. (2006). A study of teaching presence and student sense of learning community in fully online and web-enhanced college courses. Internet High. Educ. 9, 175–190. doi: 10.1016/j.iheduc.2006.06.005

[ref42] Szeto E. (2015). Community of Inquiry as an instructional approach: what effects of teaching, social and cognitive presences are there in blended synchronous learning and teaching? Comput. Educ. 81, 191–201. doi: 10.1016/j.compedu.2014.10.015

[ref43] Tóth T. Virágh R. Hallová M. Stuchlý P. Hennyeyová K. (2022). Digital competence of digital native students as prerequisite for digital transformation of education. Int. J. Emerg. Technol. Learn. 17, 150–166. doi: 10.3991/ijet.v17i16.31791

[ref44] Wang G. X. Bai C. J. Lu H. (2012). An empirical analysis of teaching presence in network distance education courses. China Educ. Technol. 9, 42–47.

[ref45] Wang W. P. Li W. (2022). Regional differences in experiences of online learning among Chinese college students and influencing factors: based on analysis of the survey data of 334 institutions of higher education. Chin. Educ. Soc. 55, 384–403. doi: 10.1080/10611932.2023.2213155

[ref46] Wang Y. Liu Q. (2020). Effects of online teaching presence on students' interactions and collaborative knowledge construction. J. Comput. Assist. Learn. 36, 370–382. doi: 10.1111/jcal.12408, PMID: 27885969

[ref47] Wang Y. Zhao L. Shen S. Chen W. (2021). Constructing a teaching presence measurement framework based on the Community of Inquiry Theory. Front. Psychol. 12:694386. doi: 10.3389/fpsyg.2021.694386, PMID: 34899457 PMC8651543

[ref48] Weinert F. E. (2001). Concept of competence: A conceptual clarification. US: Hogrefe & Huber Publishers.

[ref49] Wu M. L. (2009). Structural equation modeling: Operations and applications of AMOS. Chongqing: Chongqing People's Publishing House, 23–25.

[ref50] Wu X. G. Chen X. H. Wu J. (2017). On the effect of presence on online learning. Mod. Dis. Educ. 2, 24–30.

[ref51] Wu D. G. Li W. (2022). The interim characteristics of large-scale online education at Chinese institutions of higher education: an empirical study based on a questionnaire survey of students instructors, and administrative personnel. Chin. Educ. Soc. 55, 330–383. doi: 10.1080/10611932.2023.2213140

[ref52] Yang L. J. Zhang W. (2016). Influencing factors and mechanism of college Students' learning engagement. High. Educ. Develop. Eval. 32, 49–61.

[ref53] Zarifis A. Efthymiou L. (2022). The four business models for AI adoption in education: giving leaders a destination for the digital transformation journey. In 2022 IEEE Global Engineering Education Conference (EDUCON), 1868–1872. doi: 10.1109/EDUCON52537.2022.9766687

[ref54] Zhao H. Sullivan K. P. (2017). Teaching presence in computer conferencing learning environments: effects on interaction, cognition and learning uptake. Br. J. Educ. Technol. 48, 538–551. doi: 10.1111/bjet.12383

